# A Basic Study of the Effects of Mulberry Leaf Administration to Healthy C57BL/6 Mice on Gut Microbiota and Metabolites

**DOI:** 10.3390/metabo13091003

**Published:** 2023-09-10

**Authors:** Li Gan, Yuga Inamura, Yu Shimizu, Yuki Yokoi, Yuki Ohnishi, Zihao Song, Yasuhiro Kumaki, Takashi Kikukawa, Makoto Demura, Masaaki Ito, Tokiyoshi Ayabe, Kiminori Nakamura, Tomoyasu Aizawa

**Affiliations:** 1 Laboratory of Protein Science, Graduate School of Life Science, Hokkaido University, Sapporo 060-0810, Hokkaido, Japan; 2 Laboratory of Biological Information Analysis Science, Faculty of Advanced Life Science, Hokkaido University, Sapporo 060-0810, Hokkaido, Japan; 3 Innate Immunity Laboratory, Faculty of Advanced Life Science, Hokkaido University, Sapporo 001-0021, Hokkaido, Japan; 4 National Institute of Technology, Okinawa College, Nago 905-2192, Okinawa, Japan

**Keywords:** mulberry leaf, C57BL/6 mice, gut microbiota, NMR spectroscopy, metabolomics

## Abstract

Mulberry leaves contain α-glucosidase inhibitors, which have hypoglycemic effects and are considered functional foods. However, few reports have covered the effects of mulberry leaf components on normal gut microbiota and gut metabolites. Herein, gut microbiota analysis and NMR-based metabolomics were performed on the feces of mulberry leaf powder (MLP)-treated mice to determine the effects of long-term MLP consumption. Gut microbiota in the mouse were analyzed using 16S-rRNA gene sequencing, and no significant differences were revealed in the diversity and community structure of the gut microbiota in the C57BL/6 mice with or without MLP supplementation. Thirty-nine metabolites were identified via ^1^H-NMR analysis, and carbohydrates and amino acids were significantly (*p* < 0.01–0.05) altered upon MLP treatment. In the MLP-treated group, there was a marked increase and decrease in maltose and glucose concentrations, respectively, possibly due to the degradation inhibitory activity of oligosaccharides. After 5 weeks, all amino acid concentrations decreased. Furthermore, despite clear fluctuations in fecal saccharide concentrations, short-chain fatty acid production via intestinal bacterial metabolism was not strongly affected. This study provides the knowledge that MLP administration can alter the gut metabolites without affecting the normal gut microbiota, which is useful for considering MLP as a healthy food source.

## 1. Introduction

The mulberry tree, which is classified in the genus *Morus*, is extensively cultivated in Japan, China, Korea, Thailand, and several other Asian countries, and its dried leaves are used as tea leaves. Mulberry tea has long been considered a Chinese medicine and a functional food in Asian countries [1]. A suspension of fine powder or liquid extracted with hot water is consumed as tea. Previous studies indicated that mulberry leaves are rich in bioactive compounds, including flavonoids, alkaloids, polysaccharides, polyphenols, and volatile oils, while also containing a wealth of amino acids, various inorganic trace elements, and vitamins [2,3,4]. These constituents confer upon mulberry leaves a range of functions contributing to health, including anti-hyperglycemic, anti-hyperlipidemic, anti-obesity, antioxidant, and anti-inflammatory effects [2,3,5,6]. Among these, reports of its strong anti-diabetic effects are increasing and attracting attention. For example, it has been pointed out that the onset of diabetes is related to an increase in blood glucose levels due to the accumulation of lipid peroxide caused by active oxygen radicals. Flavonoids contained in mulberry leaves were reported to inhibit this oxidative stress and prevent diabetes caused by high blood glucose. In addition, it was also reported that flavonoids and polyphenols from mulberry leaves effectively improve insulin resistance, which is one of the causes of diabetes.

Among the constituents of mulberry leaves involved in diabetes inhibition, 1-deoxynojirimycin (1-DNJ) is a well-known bioactive compound unique to mulberries [2]. 1-DNJ is an iminosugar, a glucose analog in which the oxygen atom of the pyranose ring is replaced with an NH group [7]. Several studies have reported that 1-DNJ is a potent α-glucosidase inhibitor [7,8] that controls blood glucose levels [8] and improves insulin sensitivity [9]. 1-DNJ competitively binds to the catalytic center of α-glucosidase, thereby impeding the entry of substrates, while inducing conformational changes in the enzyme molecule, effectively inhibiting the catalytic activity of α-glucosidase [10]. Owing to these effects, 1-DNJ and mulberry leaves are considered promising therapeutic approaches for treating type 2 diabetes mellitus (T2DM). Considering the increasing prevalence of T2DM patients worldwide [11], it is important to investigate the effects of mulberry leaves on animals.

Several previous studies have demonstrated the effects of mulberry leaves on animal metabolism by measuring changes in metabolic indicators through in vivo experiments [12], detecting the effects of mulberry leaf extracts on specific gene expression and protein synthesis using molecular biology techniques [13,14], and evaluating the effects of mulberry leaf extracts on blood glucose control in patients with diabetes through clinical trials [15,16]. Furthermore, 1-DNJ in mulberry leaves was reported to not only inhibit glucose absorption in the intestine by suppressing polysaccharide degradation but also downregulate the mRNA and protein expression of glucose transporters in the gut [17]. Other studies investigated the effects of mulberry leaf extracts on the gut microbiota, short-chain fatty acids, and branched-chain amino acids in the feces of disease-model mice [13,18,19].

However, to the best of our knowledge, no study has directly examined the inhibition of polysaccharide degradation in fecal samples, which may be important with regard to the primary mechanism of action of 1-DNJ. In particular, the strong α-glucosidase inhibitory activity of 1-DNJ may not only inhibit saccharide absorption in the host small intestine but also provide a source of nutrients to the microbiota in the digestive tract. Furthermore, several studies examined the effects of the mulberry leaf extract and 1-DNJ on the gut microbiota, and these studies focused on the effects in disease-model mice [20,21]. Considering that mulberry leaf tea is consumed daily as a luxury or functional food, knowledge of the gut microbiota and metabolites in the normal host intestine is important.

Therefore, this study examined the effects of a mulberry leaf powder (MLP) suspension administered to healthy mice for a relatively long period of 9 weeks on gut microbiota and metabolites. The composition of the gut microbiota was analyzed using 16S-rDNA sequencing. Furthermore, a nuclear magnetic resonance (NMR)-based metabolomics approach was employed to investigate changes in water-soluble metabolites, including saccharides, in the feces of MLP-fed mice.

## 2. Materials and Methods

### 2.1. Animal Treatment

A total of 10 male C57BL/6J mice (6 weeks old) were purchased from CLEA Japan, Inc. (Tokyo, Japan). The mice were individually housed in cages and maintained under controlled conditions (25 ± 2 °C, 50–70% humidity, and a 12 h light/dark cycle) with ad libitum access to water and a CE-2 diet (CLEA Japan, Inc.). All mice were acclimated for three weeks before the start of the formal experiment. The mice were randomly divided into control (*n* = 4) and MLP-treated groups (*n* = 6). All animal experiments were conducted in accordance with the Hokkaido University Regulations for Animal Experimentation, following approval from the Institutional Animal Care and Use Committee of the National University Corporation at Hokkaido University.

MLP prepared from *Morus australis* was purchased from Urasoe City Silver Human Resources Center (Okinawa, Japan). This MLP was shown to contain approximately 4 mg/g of 1-DNJ [22]. MLP doses in this study were calculated from the standard human intake of two cups of tea per day (1.1 g/100 mL × 2 = 2.2 g). The mouse MLP dose (453 mg/kg) was converted from a human equivalent dose on the basis of body surface area using the following formula from the US Food and Drug Administration (available from https://www.fda.gov/media/72309/download, accessed on 5 September 2023): assuming a human weight of 60 kg, the human equivalent dose of 2.2 g/60 kg (36.8 mg/kg) = 36.8 × 12.3 = 453 mg/kg mouse dose, where a conversion coefficient of 12.3 was used to account for difference in body surface area between a mouse and a human. The 9-week dosing period was determined with reference to previous MLP dosing experiments [23]. MLP-treated mice were orally administered MLP suspended suspension (13.6 mg/mL, twice daily, 8 A.M. and 8 P.M., 500 µL each time) for 9 weeks using a disposable oral sonde (Fuchigami, Japan). Mice in the control group were orally administered the same volume of double-distilled water daily for 9 weeks using a disposable oral sonde. The body weights of all mice were recorded daily at 8 A.M.

Mouse feces were collected at 8 A.M. once per week. After collection, the feces were stored promptly at –80 °C. The mice were subjected to a fasting period of five hours starting at 9 A.M. on the same day. Blood was obtained from the tail at 2 P.M., and glucose concentrations were measured using a LAB Gluco (ForaCare, Tokyo, Japan) at weekly intervals.

### 2.2. Fecal DNA Extraction and 16S-rRNA Sequencing

Total DNA was extracted from 200 mg of the fecal sample using the QIAamp Fast DNA Stool Mini Kit (QIAGEN, Hilden, Germany), following the manufacturer’s instructions. The DNA concentration was determined by measuring the absorbance at 260 nm using a NanoDrop 2000 spectrophotometer (Thermo Fisher Scientific, Waltham, MA, USA).

Amplification of the 16S-rRNA genes from fecal DNA extracts was performed using the universal primer sets Bakt 341F (5-cctacgggnggcwgcag) and Bakt 805R (5-gactachvgggtatctaatcc) [24]. The PCR reaction was conducted in a 25 μL reaction mixture containing 0.5 ng/μL of template DNA, 200 nM of each universal primer, and 1 × KAPA HiFi Hot Start Ready Mix (Kapa Biosystems, Wilmington, MA, USA). The amplification protocol consisted of an initial denaturation step at 95 °C for 3 min, followed by 25 cycles, with each cycle consisting of 30 s at 95 °C, 30 s at 55 °C, and 30 s at 72 °C. Finally, an extension step was performed at 72 °C for 5 min. The PCR products were purified using AMPure XP beads (Beckman Coulter, Bres, CA, USA), followed by index PCR in a 50 μL reaction mixture consisting of 5 μL of PCR amplicons, 5 μL of each indexing primer containing adapter sequence and sample specific 8 bp barcodes in the Nextera XT Index Kit v2 Set B (Illumina, San Diego, CA, USA), and 1× KAPA HiFi Hot Start Ready Mix. The amplification conditions were as follows: an initial denaturation step at 95 °C for 3 min, followed by eight cycles, with each cycle consisting of 30 s at 95 °C, 30 s at 55 °C, and 30 s at 72 °C. Finally, an extension step was performed at 72 °C for 5 min. Each amplified product was quantified using a Qubit dsDNA HS assay kit (Invitrogen, Carlsbad, CA, USA) after purification, and was adjusted to a concentration of 4 nM. Subsequently, 4 µL of pooled amplicons were subjected to quantitative PCR using the KAPA Library Quantification Kit Lightcycler 480 qPCR Mix (Kapa Biosystems), followed by denaturation and dilution to 4 pM according to Illumina’s guidelines. The amplicon library was mixed with 5% of 4 pM PhiX Control v3 (Illumina) and subjected to paired-end sequencing on a MiSeq instrument using the MiSeq 600-cycle v3 kit (Illumina).

### 2.3. 16S rDNA-Based Taxonomic Analysis

After the read-quality filtering and base-calling, the obtained sequence reads were demultiplexed using the bcl2fastq software (Illumina). The FASTQ files generated by MiSeq and taxonomic analyses were performed using QIIME2 software (version 2019.7) [25]. During the quality filtering, denoising, and chimeric sequence removal using the DADA2 plugin [26], the following parameters were used: –p-trim-left-f 17, –p-trim-left-r 21, –p-trunc-len-f 280, –p-trunc-len-r 200, –p-max-ee-f 2, and –p-max-ee-r 2. Initially, the alignment was performed using MAFFT [27], followed by a phylogenetic tree construction using FastTree [28]. Using a naïve Bayes classifier, each feature was taxonomically assigned based on 99% sequence similarity using the SILVA database (v.132) [29]. α-Diversity metrics, including observed operational taxonomic units (OTUs), phylogenetic diversity (PD), whole-tree diversity, the Shannon index, and the Simpson index, were calculated using the Qiime2 pipeline. β-Diversity was assessed using the weighted UniFrac distance. The statistical significance of the β-diversity was determined using the PERMANOVA test within the Qiime2 pipeline.

### 2.4. Sample Preparation for ^1^H NMR Analysis

Fecal pellets (60–85 mg) were weighed and mixed with a 1:13 (*w*/*v*) ratio of phosphate buffer solution (50 mM Na_2_HPO_4_/NaH_2_PO_4_, pH 7.4, 10% *v*/*v* D_2_O) containing 1 mM 3-trimethylsilypropionate-2,2,3,3-d4 (TSP-d4) as a chemical shift reference (δ 0.00) and 0.04% NaN_3_. The mixture was vortexed for 30 s and shaken for 30–45 min at 4 °C. The homogenates were centrifuged at 15,000 rpm for 10 min at 4 °C. Supernatants (550 µL) were transferred into 5 mm NMR tubes.

### 2.5. NMR Spectra Acquisition and Data Processing

^1^H-NMR spectra were recorded using a Bruker 600 MHz AVANCE III spectrometer (Bruker Biospin, Rheinstetten, Germany) equipped with a TXI z-gradient probe at a proton frequency of 600.13 MHz, and the sample temperature was controlled at 298 K. The noesy1d presaturation pulse sequence was used to reduce the residual water signal with a low-power selective pulse at the water frequency during a relaxation delay of 3.5 s and a mixing time of 0.1 s. Each spectrum comprised 28,844 data points with a spectral width of 9615 Hz. The acquisition time was 1.5 s, and the number of scans was 128.

All free induction decays were zero-filled to 115 K and an exponential line-broadening function of 0.2 Hz was applied before the spectra were Fourier transformed. All spectra were manually corrected for phase and baseline distortions against TSP resonance at δ = 0.0 ppm using Delta 5.0.4 (JEOL, Tokyo, Japan). The NMR spectra were segmented into regions at 0.4–10.0 ppm with a bucket width of 0.005 ppm, excluding the water residue (4.64–5.20 ppm), to obtain binning results by using an NMR Suite 8.2 Processor (Chenomx Inc., Edmonton, AB, Canada). All metabolite assignments and quantifications were determined by referencing the 600 MHz library from the Chenomx NMR Suite 8.2 Profiler (Chenomx Inc.). Some of the NMR spectral peaks of certain metabolites may have been affected by peaks from residual water, which were quantified with reference to their less-affected portions.

### 2.6. Multivariate Analysis of the NMR Data

The NMR spectral data matrix was exported to SIMCA 15 software (Umetrics, Umeå, Sweden). Principal component analysis (PCA) and orthogonal partial least squares discriminant analysis (OPLS-DA) were used to reduce the dimensionality of the dataset. PCA and supervised classification of OPLS-DA were conducted to extract significant metabolite information. In the data preprocessing, Pareto scaling was implemented prior to the PCA and OPLS-DA for the binning result analysis. Unit variance was applied to the mean-centered data before OPLS-DA for metabolite quantification analysis. A score plot was obtained from the data to visualize the clustering pattern of fecal samples along two principal components (PC1 and PC2), where each point denoted an individual spectrum of a fecal sample. Loading plots were used to analyze the metabolites responsible for group segregation. The variable importance in projection (VIP) was obtained to indicate the overall importance of each variable.

Statistical analyses were conducted utilizing GraphPad Prism 8.4.3 (GraphPad Software, San Diego, CA, USA). Values are presented as the mean ± standard error of the mean (SEM). Student’s *t*-test was used to assess the statistical significance of the differences in metabolite concentrations between groups at each time point. A *p*-value < 0.05 was considered statistically significant. A t-test was performed to test only significant differences between the treatment and control groups at each time point.

## 3. Results

### 3.1. Body Weight and Blood Glucose

Daily body weight measurements were performed for all the mice, and the average weekly weights of the mice are shown in Table S1. No significant differences were observed in weight change between the MLP-treated and control groups.

**Figure 1:**
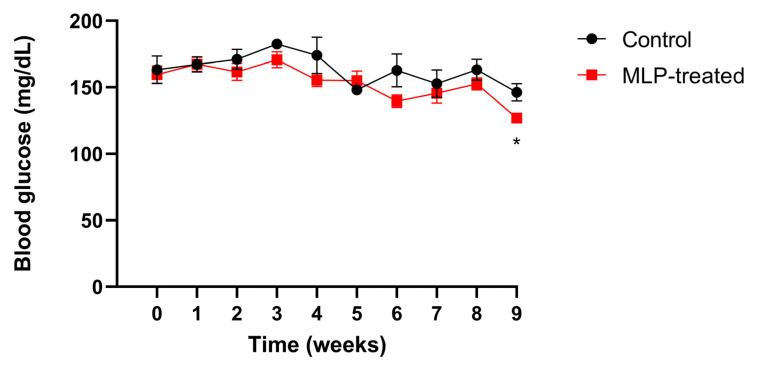
The blood glucose level of control (black) and MLP-treated (red) mice. Values are presented as mean ± SEM. The asterisk indicates a significant difference between control and MLP-treated groups (* *p* < 0.05); *p*-values were calculated using Student’s *t*-test. A *t*-test was performed to test only significant differences between the treatment and control groups at each time point.

Mice blood glucose levels were measured weekly (Figure 1). After week 3, the blood glucose levels in the MLP group were slightly lower than those in the control group, and by week 9, they were significantly lower. These results suggest that MLP had a slight suppressive effect on blood glucose levels in healthy mice.

### 3.2. Effects of MLP Treatment on the Intestinal Microbiota

**Figure 2:**
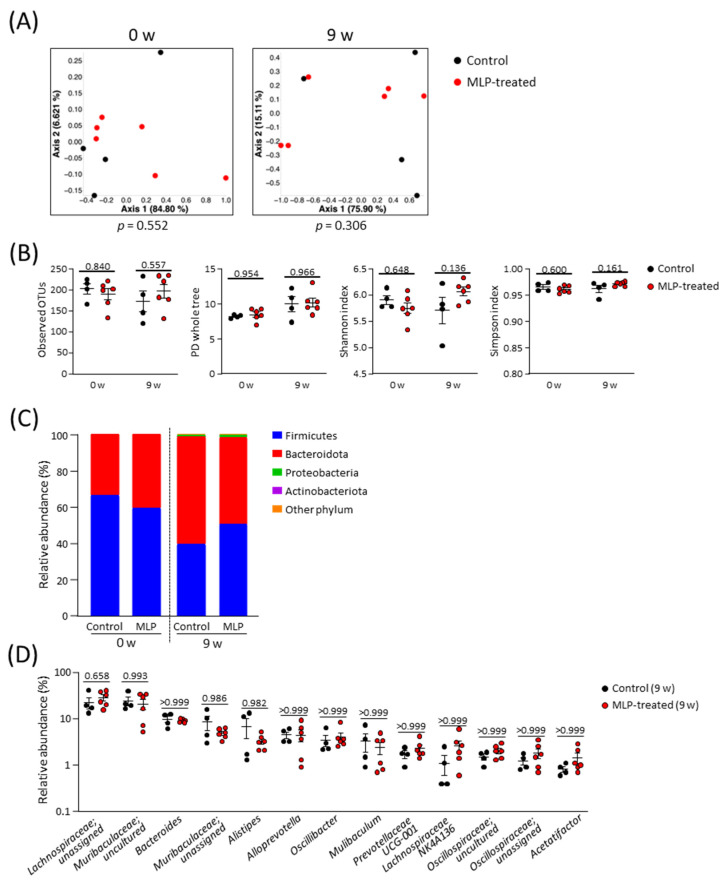
Structural composition of the gut microbiota. (**A**) PCoA plot based on weighted UniFrac distance of control and MLP-treated groups; statistical significance was evaluated using the PERMANOVA test. (**B**) Comparison of α-diversity indexes between control and MLP-treated groups at week 0 and week 9. (**C**) Stacked bar chart of relative abundance of each phylum. (**D**) Comparison of relative abundance of major genera (genera with an average of relative abundance > 1% in all mice at week 9) between groups at week 9. (**B**,**D**) Data are presented as mean ± SEM and statistical significance was evaluated using two-way ANOVA followed by Sidak’s multiple comparison tests. A *p*-value < 0.05 was considered statistically significant.

A comparison of the β-diversity in the microbial communities between the two groups was conducted using a principal coordinate analysis (PCoA) plot based on weighted UniFrac distances (Figure 2A). These results showed no significant differences in diversity between the two groups at weeks 0 or 9. The α-diversity of the microbial community was evaluated using the whole PD tree, OTUs, Shannon index, and Simpson index (Figure 2B). Because there were no significant differences between the two groups at 9 weeks, we concluded that the MLP treatment did not affect the diversity of the intestinal microbiota in healthy mice.

No significant differences were observed at the phylum level between the control and MLP-treated groups at weeks 0 and 9 (Figure 2C, Figure S1). Over time, the relative abundances of Bacteroidota and Firmicutes decreased in both the control and MLP-treated groups. Proteobacteria and Actinobacteria were detected with only slight relative abundances in both groups at 9 weeks. For a more detailed analysis, the gut microbiota compositions of both groups at week 9 were compared at the genus level (Figure 2D). Among the 13 most abundant genera, no genera showed significant differences between the control and MLP-treated groups. Taken together, these results indicate that the 9-week MLP treatment had no significant effect on the intestinal microbiota.

### 3.3. ^1^H-NMR Spectra of Mice Feces and Identification of Metabolites

**Figure 3:**
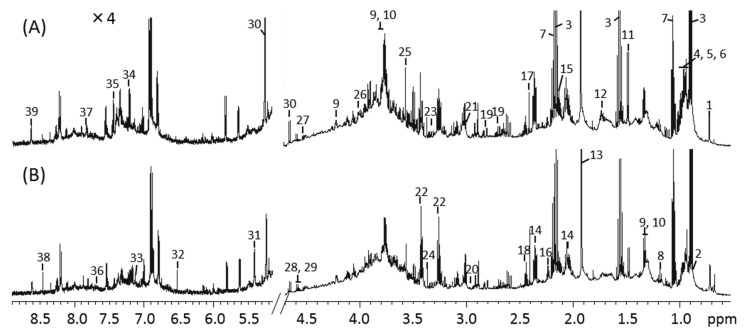
^1^H-NMR spectra of mouse fecal extraction obtained from (**A**) control and (**B**) MLP-treated mice. The 5.2–8.9 ppm region is magnified 4× compared with the corresponding region of 0.5–4.7 ppm. Numbers indicate the following metabolites: 1, Cholate; 2, Valerate; 3, Butyrate; 4, Isoleucine; 5, Leucine; 6, Valine; 7, Propionate; 8, Ethanol; 9, Threonine; 10, Lactate; 11, Alanine; 12, Lysine; 13, Acetate; 14, Glutamate; 15, Methionine; 16, 5-aminopentanoate; 17, Succinate; 18, Glutamine; 19, Aspartate; 20, Asparagine; 21, 2-Oxoglutarate; 22, Taurine; 23, Proline; 24, Methanol; 25, Glycine; 26, Serine; 27, Arabinose; 28, Xylose; 29, Galactose; 30, Glucose; 31, Maltose; 32, Fumarate; 33, 4-hydroxyphenylacetate; 34, Tyrosine; 35, Phenylalanine; 36, Tryptophan; 37, 4-Hydroxybenzoate; 38, Formate; 39, Nicotinate.

The ^1^H NMR spectra of mouse feces from the control and MLP-treated groups at week 9 are shown in Figure 3. Using the Chenomx library, 39 metabolites were identified from the NMR spectra. These included amino acids (e.g., alanine, valine, leucine, isoleucine, serine, tyrosine, and proline), organic acids (e.g., butyrate, propionate, acetate, valerate, lactate, and succinate), and carbohydrates (e.g., glucose and maltose). Other metabolites, such as cholate, nicotinate, and ethanol, were also identified.

### 3.4. Multivariate Analysis of Metabolites

**Figure 4:**
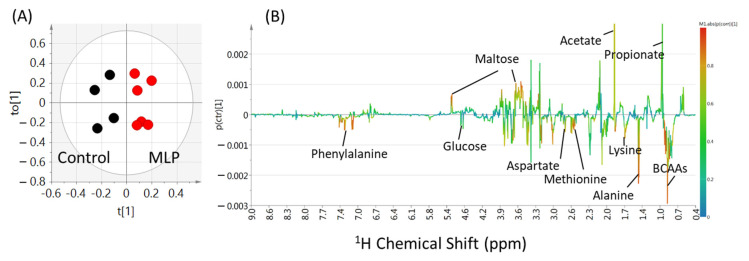
OPLS-DA score plot (**A**) and loading plot (**B**) of feces samples from control (black) and MLP-treated mice (red) at week 9. The levels of fecal metabolites in the MLP-treated mice were increased and decreased corresponding to the upward and downward pointing peaks, respectively, observed in the NMR spectra. Metabolites represented by warmer colors in the spectrum contributed more significantly to the separation than metabolites represented by cooler colors. Two components, R^2^X = 0.621, R^2^Y = 0.876, Q^2^ = 0.736.

To clarify the differences between the control and MLP-treated groups at 0–9 weeks, an OPLS-DA multivariate statistical analysis was performed on the binning values of the NMR spectra. The R^2^X, R^2^Y, and Q^2^ values are listed in Table S2. Higher values of Q^2^ indicated higher model reliability. The Q^2^ values were lower than 0 at 0-3 weeks, while higher than zero after 4 weeks. At 9 weeks, the PCA score plots did not show good separation (Figure S2, three components, R^2^X = 0.817, Q^2^ = 0.476), whereas the PLS-DA model score plots showed clear separation (Figure S3; two components, R^2^X = 0.621, R^2^Y = 0.876, Q^2^ = 0.712). In the OPLS-DA model, the score plot showed further discrimination between the control and MLP-treated groups (Figure 4A; 1+1+0 components, R^2^X = 0.621, R^2^Y = 0.876, Q^2^ = 0.736). In the OPLS-DA coefficient loading plot, significant signals for discrimination between the control and MLP-treated groups (warmer colors; Figure 4B) were observed. Based on the chemical shifts, compounds including amino acids (alanine and BCAAs), organic acids (acetate and propionate), and carbohydrates (glucose and maltose) were shown to contribute to this separation.

To further clarify the contribution of each metabolite, their concentrations were quantified from the NMR spectra. Analysis of the quantitative results of the metabolites at week nine revealed a clear separation between the two groups in the OPLS-DA score plot (Figure S4A; one component, R^2^X = 0.71, R^2^Y = 0.842, Q^2^ = 0.694). By examining the loading plot (Figure S4B), the metabolites that primarily contributed to the separation of the two groups were determined. Consistent with the results of the non-target analysis using binning values, amino acids, maltose, and branched-chain amino acids ranked in the top three in the VIP plot (Figure S4C).

### 3.5. Comparison of Metabolite Quantitative Value Variability

**Figure 5:**
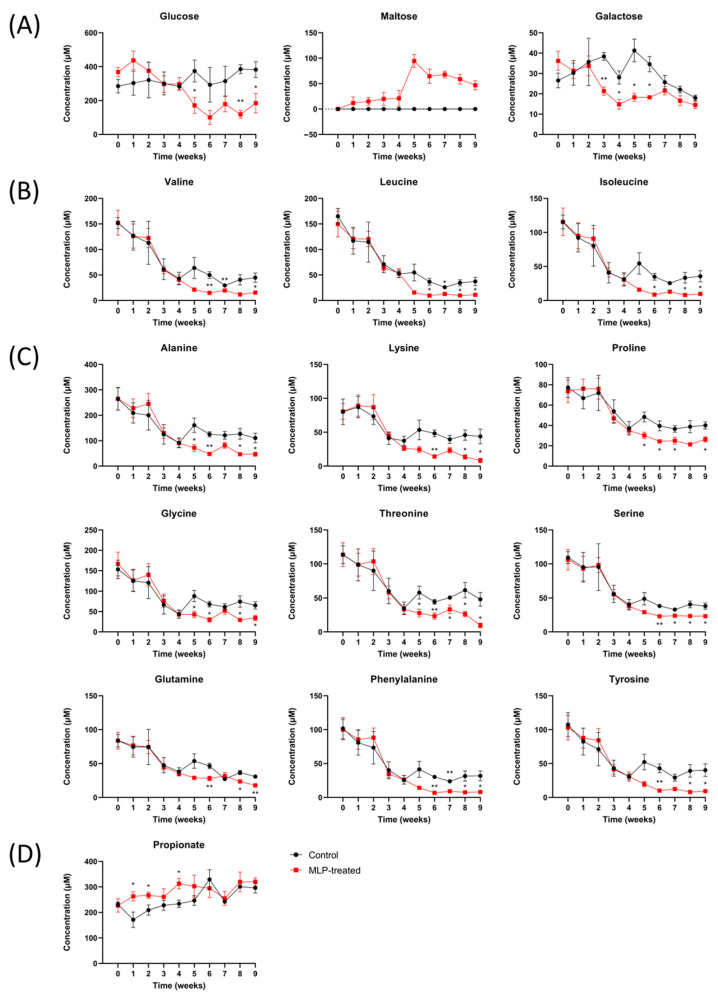
Time course of the metabolite levels in control (black line) and MLP-treated (red line) mouse feces. (**A**) Carbohydrates. (**B**) Branched-chain amino acids. (**C**) Other amino acids. (**D**) Short-chain fatty acids. Values are presented as mean ± SEM. Asterisk indicates a significant difference between control and MLP-treated mice feces (* *p* < 0.05, ** *p* < 0.01); *p*-values were calculated using Student’s *t*-test. A *t*-test was performed to test only significant differences between the treatment and control groups at each time point.

Figure 5 shows the quantitative time-course values of significantly increasing and decreasing metabolites compared with the control group based on quantitative analysis using Chenomx and Student’s *t*-test. The other metabolites are shown in Figure S5, and all *p*-values of fecal metabolites between the control and MLP-treated groups are shown in Table S3. 

First, regarding carbohydrates (Figure 5A), maltose was detected in only the MLP-treated group and not in the control group over the entire period. In the MLP-treated group, maltose was detected beginning at week 1, showing a sharply higher value at week 5, and then gradually decreased again. These results suggest that α-glucosidase, which degrades maltose, was more strongly inhibited than α-amylase, which degrades starch in feed to produce maltose. Furthermore, MLP-treated mice showed significantly lower concentrations of glucose than the control mice from week 5, whereas the maltose concentrations increased sharply. Galactose showed a significant decrease from week 3 in the MLP-treated group, recovered at week 7, and reached the same level as that of the control group. 

Next, the amino acid concentrations between the two groups were significantly different after week 5 (Figure 5B,C). In addition to the branched-chain fatty acids of valine, leucine, and isoleucine (Figure 5B), concentration changes with very similar characteristics were observed for alanine, lysine, proline, lysine, threonine, serine, glutamine, phenylalanine, and tyrosine (Figure 5C). In other words, the MLP-treated group showed similar changes to the control group from weeks 0 to 4; however, after week 5, the common characteristic was a significantly lower concentration. This feature was also observed for other amino acids, such as aspartic acid, glutamic acid, and methionine, although the difference was not significant (Figure S5).

Finally, the concentration of propionic acid, which is a typical short-chain fatty acid, was significantly higher in the MLP group than in the control group at weeks 1–4 (Figure 5D). After week 5, there were no significant differences between the two groups. In contrast, no significant differences were observed for short-chain fatty acids, such as formic acid, acetic acid, and butyric acid, or for organic acids, such as lactic acid and succinic acid (Figure S5).

## 4. Discussion

The global prevalence of T2DM, its side effects, and the high cost of drugs required for its treatment are reasons to explore safer and more reliable herbal therapies [30,31,32,33]. Mulberry leaves, which are a traditional Chinese herbal medicine, have been considered a potential treatment for T2DM owing to their ability to reduce glucose absorption [1]. Mulberry leaf flavonoids, alkaloids, polysaccharides, and polyphenols were reported as the primary molecular basis for lowering blood sugar levels [34]. The molecular mechanisms underlying their blood-glucose-regulating effects are extremely diverse and are thought to result from improvements in oxidative stress, insulin resistance, lipid metabolism, and gut microbiota [34]. Among the useful compounds in mulberry leaves, 1-DNJ, which is an alkaloid, is a well-studied inhibitor of elevated blood glucose in terms of its mechanism of action in inhibiting the degradation of polysaccharides through the inhibition of α-glucosidase and α-amylase [7,8,34]. In recent years, the action of 1-DNJ absorption into the bloodstream to improve insulin sensitivity has attracted attention; however, the [9] inhibition of polysaccharide breakdown and absorption in the gastrointestinal tract is undoubtedly one of the primary mechanisms for the inhibition of blood glucose elevation. Furthermore, the flavonoids of mulberry leaves also have α-glucosidase inhibitory activity. Flavonoids, such as rutin, isoquercitrin, kaempferol-3-0-rutinoside, astragaloside, and sapogenins, in extracts of mulberry leaves showed α-glucosidase-inhibitory activity [35]. In this study, we succeeded in obtaining several new findings from analyses focusing on the gut microbiota and metabolites in feces, which are thought to be directly and most significantly affected by the suppression of degradation of these polysaccharides. Mulberry leaves are consumed not only as an herbal medicine but also as a luxury food, such as tea, or as a health food [7,8,34]. Therefore, herein, we employed an experimental design using healthy mice, rather than disease-model mice, to obtain basic knowledge on the effects on normal gut microbiota and metabolites. Apart from reports on the safety and toxicity of mulberry leaves [36], little is known about the effects of mulberry leaf consumption on the healthy intestinal environment, which was the focus of this study.

First, the main site where the degradation inhibitory activity of mulberry leaves on polysaccharides occurs is generally considered to be the small intestine. Various components of mulberry leaves, including 1-DNJ, inhibit α-glucosidase activity in the small intestinal mucosa, thereby inhibiting the degradation of maltose to glucose and its absorption in the small intestine [34]. Second, the possibility that the α-glucosidase inhibitory activity of MLP may act on intestinal bacteria in the large intestine should not be ignored [37]. If carbohydrates are not digested and absorbed properly in the small intestine, they flow into the large intestine, where they undergo microbial fermentation [38]. For example, dietary starch, known as resistant starch, reaches the large intestine undigested and is not absorbed in the small intestine, where it serves as an energy source for intestinal bacteria [39,40]. In addition, when MLP suppresses polysaccharide breakdown in the small intestine, the amount of polysaccharides reaching the large intestine is expected to increase. α-glucosidases are also commonly found in microorganisms of the human gut microbiota, and it was suggested that α-glucosidase inhibitors may cross-react in vivo with bacterial α-glucosidases present in the gut microbiota [41]. Indeed, previous studies have revealed a high degree of sequence and structural homology in the α-glucosidase enzymes between the human intestinal α-glucosidase and the microbial α-glucosidase from *Blaubia obeum* [37]. Thus, the inhibition of these gut microbiota-derived enzymes by MLP and their effects on growth and metabolite production should be carefully discussed.

Given this background, the fact that maltose in the feces, which was completely undetected in the control group, was detected in the MLP-treated group at week 1 is a crucial finding (Figure 5). First, the presence of maltose in feces indicates that maltose production occurred, but further degradation did not occur, indicating that the inhibition of α-glucosidase, mainly DNJ-1, by MLP was stronger than the inhibition of α-amylase activity. This result is consistent with previous findings showing that 1-DNJ also has inhibitory activity against α-amylase, but is less potent than its inhibitory activity against α-glycosidase [42,43,44]. Furthermore, it is likely that maltose, which may have been produced in the upper gastrointestinal tract and small intestine, was not efficiently degraded or utilized by bacteria in the colon and was excreted in the feces. This could be due to α-glucosidase inhibitory activity against bacteria in the colon or to the fact that bacteria in the colon are less likely to utilize maltose in the first place. Interestingly, the MLP-treated group showed a sharp increase in fecal maltose concentrations at week 5, at which point, a significant decrease in glucose concentration was observed. It is clear from the results of the gut microbiota analysis in this study that the inhibition of degradation after week 5 was not due to changes in the healthy normal gut microbiota. Therefore, although it is not yet clear from this study alone, it is possible that the inhibition of α-glucosidase activity increased after 5 weeks due to some action. Almost all amino acid concentrations, including BCAAs, in the feces of the MLP-treated group decreased, indicating a large variation in gut metabolites. It is also interesting to note that α-glucosidase inhibition was observed in this study despite the fact that MLP was administered twice daily at times other than during feeding. This suggests that MLP may be able to exert its effects persistently, even if it is ingested at times unrelated to meals, for instance, as tea or other luxury items.

Short-chain fatty acids (SCFAs) are generally considered closely related to the variability of saccharides in feces. This is because SCFAs in feces are produced by intestinal bacteria fermentation and carbohydrate digestion [45]. However, despite the large differences in fecal saccharide concentrations after 5 weeks observed in this study, no significant differences were observed in major SCFAs at high concentrations, such as acetic acid and butyric acid (Figure S5). The MLP group had significantly higher levels of propionic acid, which was present at relatively high concentrations, from weeks 1 to 4, but the difference was not large (Figure 5D). These results also suggested that the administration of MLP with α-glucosidase inhibitory activity had little effect on SCFA production in normal gut microbiota. Apart from its α-glucosidase inhibitory effect, MLP is also a source of dietary fibers, such as cellulose, which contains β-glycosidic bonds [46]; however, in this study, MLP had no effect on SCFA production in this regard. Thus, the small amount of soluble fiber in the normal consumption of MLP may not have been sufficient to cause significant changes in the production of SCFAs in the normal gut microbiota.

The broad, significant, and substantial reduction in the overall amino acid concentrations after week 5 due to MLP administration may have affected the host (Figure 5B,C). In general, possible factors contributing to the decreased amino acid content in the feces include decreased proteolysis, increased uptake of amino acids from the host intestine, and utilization of amino acids as a nutrient source by intestinal bacteria owing to decreased carbohydrate concentrations [47]. Based on the fact that the decrease in amino acid concentrations in this study was synchronous with the decrease in glucose concentrations in the feces and that this decrease in amino acid concentrations occurred simultaneously for almost all amino acids, it is speculated that the utilization of amino acids as a nutrient source by intestinal bacteria may be responsible for their reduced concentration. Among these amino acids, BCAAs (Figure 5B), including valine, leucine, and isoleucine, are critical metabolic products of the gut microbiota and their levels typically increase in obese hosts [48]. Prior research demonstrated that BCAAs can inhibit the function of β-cells in regulating insulin secretion; hence, BCAAs are closely associated with insulin resistance and the risk of developing T2DM [49]. Since mammalian BCAAs are derived solely from diet and gut bacterial metabolism, understanding the effects of MLP on BCAA production in the gut microbiota is important [50]. In mice treated with MLP, the levels of BCAAs in the feces were lower than those in the control group starting from week 5, with significant differences appearing at week 6. In previous studies on T2DM mice fed high-fat and high-sugar diets, it was reported that the administration of aqueous extracts of mulberry leaves causes changes in the gut microbiota that reduce BCAA concentration and host uptake in the feces, which ultimately promotes the tissue-specific expression of BCAA catabolic enzymes in the host [19]. It is noteworthy that the mulberry leaf extract in that study did not contain 1-DNJ, and the decreased synthesis of BCAAs via the improvement of gut microbiota was thought to be the result of a different mechanism than that in this study [19]. It is interesting to note that even in the healthy mice herein, the long-term administration of mulberry leaves reduced BCAA concentrations in the intestine via a mechanism that was not mediated by effects on the gut microbiota. This is an intriguing finding when considering the ameliorative effects of mulberry leaves on T2DM owing to a combination of factors. A study using an ethanol extract of mulberry leaf, which is high in polyphenols, flavonoids, and alkaloids, reported changes in the gut microbiota in a rat model of type 2 diabetes. Compared with that study, our study, which showed no change in gut microbiota, suggests that it may only be effective in improving disrupted gut microbiota in disease models, although the possibility that simply a low dose was used cannot be completely ruled out [51]. In addition, the present study is insufficient to examine the physiological effects of these intestinal metabolite variations on the host side. In the future, it is important to examine the effects of various metabolites taken up by the host on the host based on the variation of metabolites in the blood and organs.

In conclusion, the long-term ingestion of MLP altered the concentrations of saccharides and amino acids in feces without affecting the normal gut microbiota. Recently, many studies on mouse models of disease reported that the administration of MLP and components derived from mulberry leaves can improve gut microbiota and result in favorable effects on the host. The results of this study suggest that the inhibitory activity of MLP on polysaccharide degradation may not adversely affect the normal intestinal microbiota but may improve the composition of metabolites produced by the intestinal microbiota and maintain health via pathways other than those that cause changes in the gut microbiota. The results of the present study on gut microbiota and their metabolite variation in healthy mice are important as a basis for future studies on their effects on the host.

## Data Availability

The data presented in this study are available upon request from the corresponding author. The data are not publicly available due to privacy concerns.

## Supplementary Materials

The following supporting information can be downloaded at: https://www.mdpi.com/article/10.3390/metabo13091003/s1, Table S1: Average weekly weight of mice; Table S2: Evaluation of the goodness of fit of the OPLS-DA models from the binning results; Table S3: All *p* values of fecal metabolites between the control and MLP-treated groups; Figure S1: Comparison of the relative abundance of each phylum between the groups at weeks 0 and 9. Data are presented as means  ±  SEM, and statistical significance was evaluated by two-way ANOVA followed by Sidak’s multiple comparison tests. *p* < 0.05 was considered statistically significant; Figure S2: PCA score plot of mouse feces from the control (black) and MLP-treated (red) groups at week 9. (Three Components, R^2^X = 0.817, Q^2^ = 0.476); Figure S3: PLS-DA score plot of mouse feces k from the control (black) and MLP-treated (red) groups at week 9. (Two components, R^2^X = 0.621, R^2^Y = 0.876, Q^2^ = 0.712);Figure S4. OPLS-DA score plot (A), loading plot (B), and VIP plot (C) of fecal samples from control (black) and MLP-treated (red) mice at week 9. (One Component, R^2^X = 0.71, R^2^Y = 0.842, Q^2^ = 0.694); Figure S5: Time-course quantitative values of other metabolites in control (black line) and MLP-treated (red line) mouse feces. Values are presented as means ± SEM. The asterisk indicates a significant difference between the control and MLP-treated groups (* *p* < 0.05), and *p* values were calculated using the Student’s *t*-test.
