# Supplementary material for: A Basic Study of the Effects of Mulberry Leaf Administration to Healthy C57BL/6 Mice on Gut Microbiota and Metabolites
Source: Metabolites. 2023 Sep 10;13(9):1003. doi: 10.3390/metabo13091003 (PMC10535692; doi:10.3390/metabo13091003)
Supplement: Supplementary file 1 [file metabolites-13-01003-s001.zip › metabolites-2581530-supplementary.pdf]

Supplementary Materials

Table S1. Average weekly weight of mice.
Table S2. Evaluation of the goodness of fit of the OPLS-DA models from the binning results.
Table S3. All *P* values of fecal metabolites between the control and MLP-treated groups.

Figure S1. Comparison of the relative abundance of each phylum between the groups at weeks 0 and 9. Data are presented as means ± SEM, and statistical significance was evaluated by two-way ANOVA followed by Sidak's multiple comparison tests. $P < 0.05$ was considered statistically significant.
Figure S2. PCA score plot of mouse feces from the control (black) and MLP-treated (red) groups at week 9. (Three Components, $R^2X = 0.817$, $Q^2 = 0.476$).
Figure S3. PLS-DA score plot of mouse feces k from the control (black) and MLP-treated (red) groups at week 9. (Two components, $R^2X = 0.621$, $R^2Y = 0.876$, $Q^2 = 0.712$).
Figure S4. OPLS-DA score plot (A), loading plot (B), and VIP plot (C) of fecal samples from control (black) and MLP-treated (red) mice at week 9. (One Component, $R^2X = 0.71$, $R^2Y = 0.842$, $Q^2 = 0.694$).
Figure S5. Time-course quantitative values of other metabolites in control (black line) and MLP-treated (red line) mouse feces. Values are presented as means ± SEM. The asterisk indicates a significant difference between the control and MLP-treated groups ($^*P < 0.05$), and *P* values were calculated using the Student's *t*-test.

Table S1. Average weekly weight of mice.

Time (weeks)	**Body Weight (grams; mean ± standard deviation)**	
	Control group	MLP-treated group
0	22.9 ± 0.5	22.0 ± 0.9
1	23.1 ± 0.5	22.2 ± 0.9
2	23.5 ± 0.5	22.5 ± 0.9
3	23.7 ± 0.6	22.7 ± 1.0
4	24.2 ± 0.6	23.0 ± 1.1
5	24.2 ± 0.6	23.1 ± 1.2
6	24.7 ± 0.6	23.5 ± 1.1
7	24.7 ± 0.7	23.7 ± 1.2
8	24.8 ± 0.8	24.0 ± 1.0
9	25.1 ± 0.8	24.1 ± 0.9

Table S2. Evaluation of the goodness of fit of OPLS-DA models from binning results.

Week	R^2X	R^2Y	Q^2	Number of Components
0	0.411	0.474	-0.156	1+0+0
1	0.318	0.612	-0.238	1+0+0
2	0.594	0.23	-0.117	1+0+0
3	0.356	0.401	-0.125	1+0+0
4	0.325	0.66	0.322	1+0+0
5	0.264	0.685	0.182	1+0+0
6	0.267	0.888	0.492	1+0+0
7	0.409	0.572	0.245	1+0+0
8	0.3	0.768	0.5	1+0+0
9	0.322	0.71	0.522	1+0+0

Table S3. All *P* values of fecal metabolites between the control and MLP-treated groups.

	**0w**	**1w**	**2w**	**3w**	**4w**	**5w**	**6w**	**7w**	**8w**	**9w**
2-Oxoglutarate	0.71	0.55	0.39	0.42	0.79	0.91	0.18	0.32	0.43	0.72
4-Hydroxybenzoate	0.07	0.38	0.07	0.59	<0.05	0.60	0.60	0.65	0.14	0.36
4-Hydroxyphenylacetat	0.57	<0.05	0.99	0.16	<0.1	0.19	0.71	0.48	<0.1	0.18
5-Aminopentanoate	0.57	0.95	0.32	0.48	0.46	0.36	0.84	0.59	0.95	0.70
Acetate	<0.05	0.31	<0.1	0.34	<0.1	0.48	0.81	0.42	0.79	0.25
Alanine	0.99	0.74	0.55	0.91	0.97	<0.05	<0.05	<0.1	<0.05	<0.05
Arabinose	0.84	0.22	0.75	0.96	<0.1	0.44	0.78	0.33	0.85	0.35
Asparagine	0.59	0.71	0.91							
Aspartate	0.50	<0.05	0.53	0.62	0.31	<0.1	0.14	0.14	<0.1	<0.05
Butyrate	0.34	0.52	0.32	0.21	0.13	0.72	0.23	0.93	0.30	0.68
Cholate	<0.05	0.59	0.18	0.39	<0.05	0.93	0.52	0.43	0.40	1.00
Ethanol	0.29	0.42	0.27	0.17	0.91	<0.05	0.65	0.33	0.11	0.78
Formate	0.51	0.61	0.50	0.27	0.72	0.39	0.72	0.13	0.16	0.58
Fumarate	0.10	0.86	0.30	0.30	0.25	0.87	0.69	0.32	0.48	0.42
Galactose	0.14	0.89	0.89	**<0.01**	<0.05	<0.05	<0.05	0.34	0.10	0.16
Glucose	0.14	0.18	0.66	0.96	0.74	<0.05	0.16	0.23	**<0.01**	<0.05
Glutamate	0.56	0.84	0.49	0.49	0.91	0.39	<0.05	0.52	0.13	0.41
Glutamine	0.99	0.92	1.00	0.83	0.78	0.10	**<0.01**	0.44	<0.05	**<0.01**
Glycine	0.73	0.98	0.70	0.74	0.95	<0.05	**<0.01**	0.47	<0.05	<0.05
Isoleucine	0.99	0.92	0.77	0.98	0.98	<0.1	<0.05	<0.05	<0.05	<0.05
Lactate	0.38	0.58	0.32	0.58	<0.1	0.33	0.80	0.13	0.95	0.82
Leucine	0.62	0.91	0.89	0.72	0.88	<0.1	<0.05	<0.05	<0.05	<0.05
Lysine	0.98	0.92	0.55	0.73	0.21	0.14	**<0.01**	<0.1	<0.05	<0.05
Methanol	0.95	0.47	0.86	0.16	0.52	0.24	0.14	<0.05	0.18	0.89
Methionine	0.69	0.67	0.62	0.53	0.77	<0.1	<0.1	0.32	<0.1	<0.1
Nicotinate	0.65	0.39	0.69	0.34	0.66	0.94	<0.1	0.90	0.14	0.46
Phenylalanine	0.94	0.84	0.62	0.70	0.99	0.10	**<0.01**	**<0.01**	<0.05	<0.05
Proline	0.81	0.53	0.85	0.60	<0.77	<0.05	<0.05	<0.05	<0.1	<0.05
Propionate	0.87	<0.05	<0.05	0.40	<0.05	0.28	0.54	0.66	0.65	0.37
Serine	0.86	0.94	0.94	0.98	0.78	0.12	**<0.01**	<0.05	<0.05	<0.05
Succinate	0.81	0.29	0.17	0.60	0.73	0.52	0.62	0.90	0.26	0.77
Taurine	0.50	0.49	0.26	0.72	0.94	0.92	0.31	0.43	0.66	0.46
Threonine	1.00	0.99	0.71	0.93	0.89	<0.05	**<0.01**	<0.05	<0.05	<0.05
Tryptophan	0.65	0.85	0.85							
Tyrosine	0.86	0.84	0.68	0.90	0.96	<0.1	**<0.01**	<0.05	<0.05	<0.05
Valerate	0.53	0.50	0.61	0.42	0.15	0.92	0.11	0.83	<0.1	0.70
Valine	0.98	0.99	0.85	0.95	0.88	0.13	**<0.01**	**<0.01**	<0.1	<0.05
Xylose	0.63	0.17	0.83	0.99	0.20	0.57	0.48	0.96	0.68	0.46

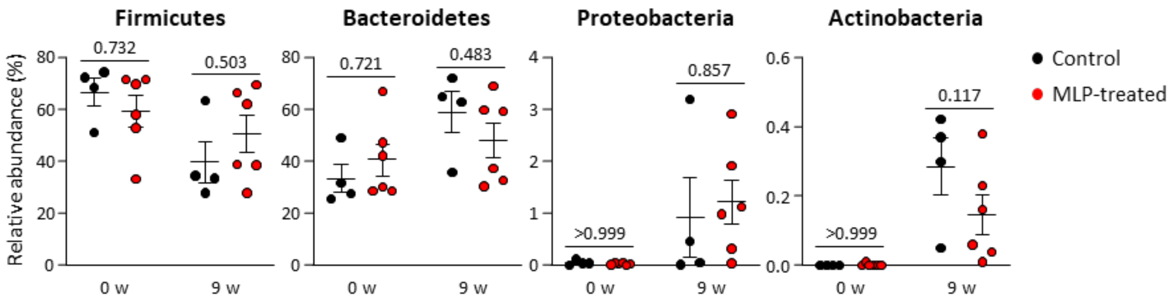

Figure S1. Comparison of the relative abundance of each phylum between the groups at weeks 0 and 9. Data are presented as means ± SEM, statistical significance was evaluated by two-way ANOVA followed by Sidak's multiple comparison tests. $P < 0.05$ was considered statistically significant.

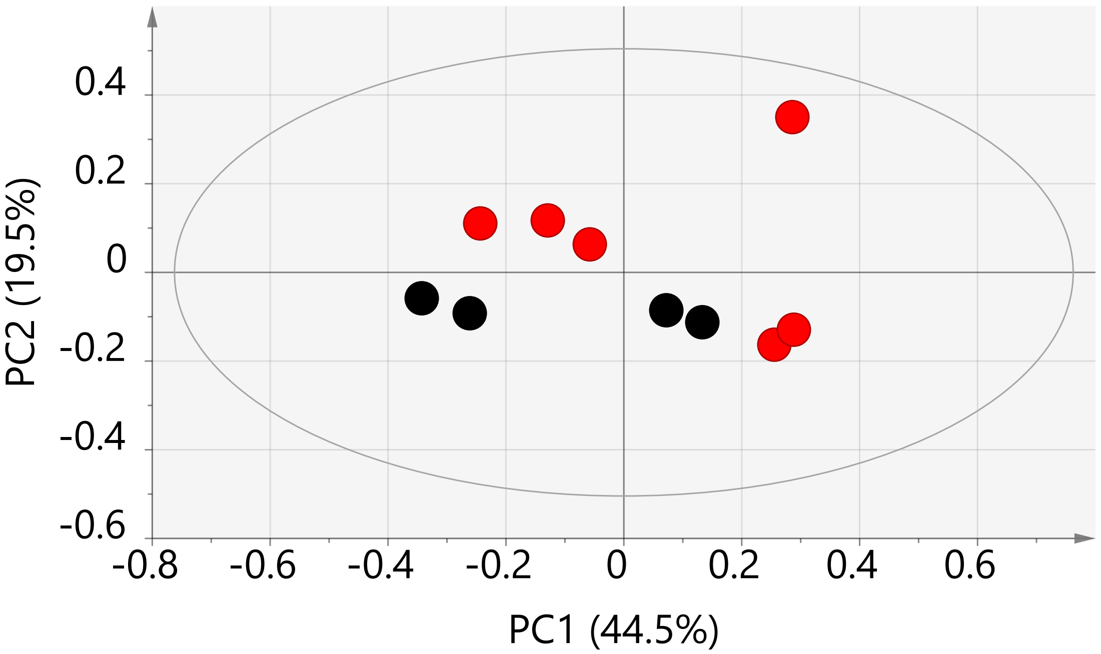

Figure S2. PCA score plot of mouse feces from the control (black) and MLP-treated (red) groups at week 9. (Three Components, $R^2X = 0.817$, $Q^2 = 0.476$).

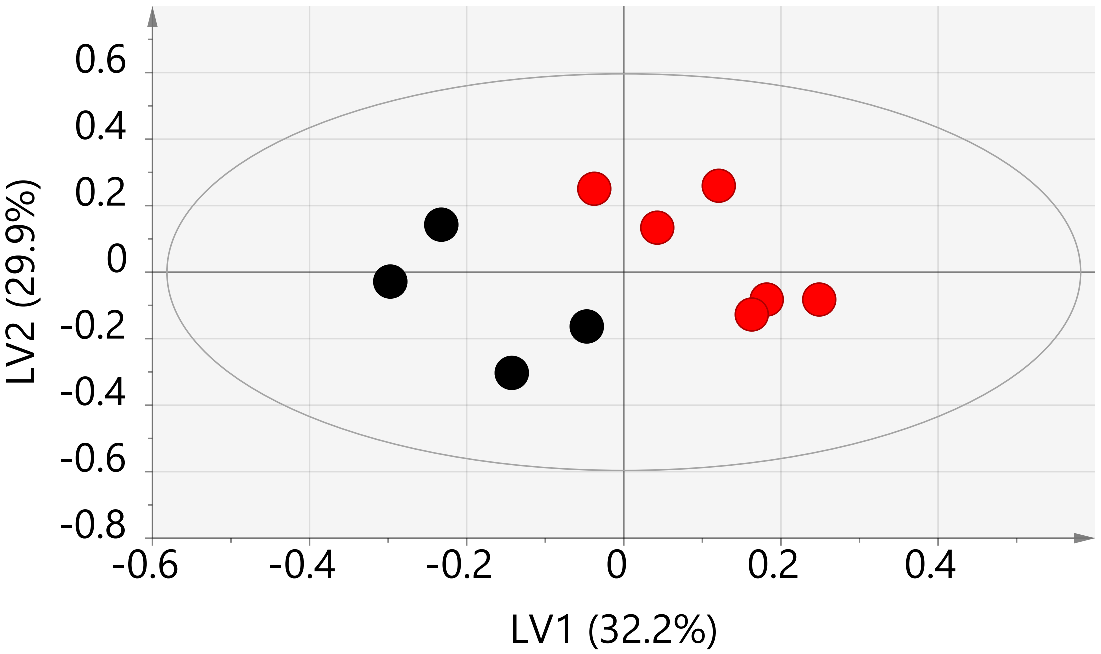

Figure S3. PLS-DA score plot of mouse feces k from the control (black) and MLP-treated (red) groups at week 9. (Two components, $R^2X = 0.621$, $R^2Y = 0.876$, $Q^2 = 0.712$).

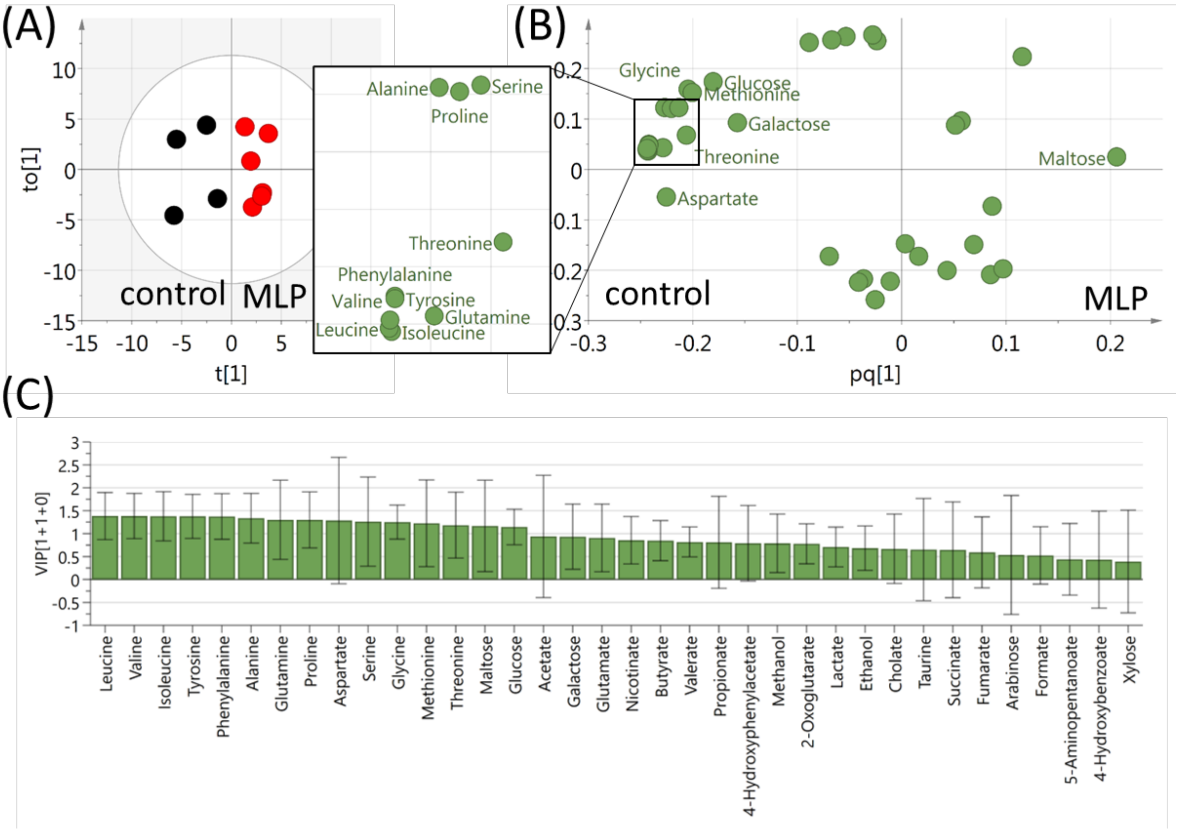

Figure S4. OPLS-DA score plot (A), loading plot (B), and VIP plot (C) of fecal samples from control (black) and MLP-treated (red) mice at week 9. (One Component, $R^2X = 0.71$, $R^2Y = 0.842$, $Q^2 = 0.694$).

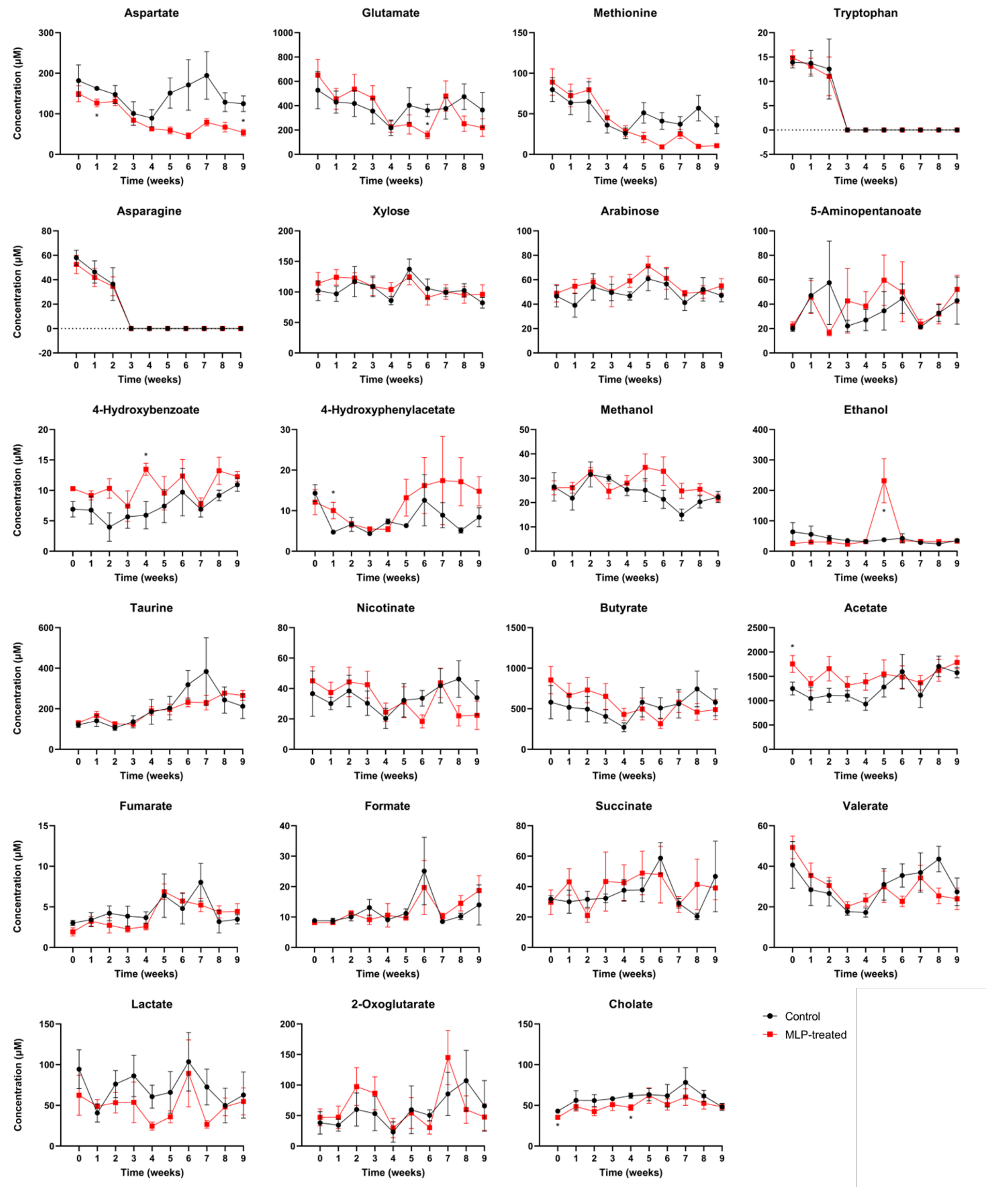

Figure S5. Time-course quantitative values of other metabolites in control (black line) and MLP-treated (red line) mouse feces. Values are presented as means ± SEM. The asterisk indicates a significant difference between the control and MLP-treated groups (*$P < 0.05$), and P values were calculated using Student's t-test.
